# Supplementary material for: Modeling glioblastoma heterogeneity as a dynamic network of cell states
Source: Mol Syst Biol. 2021 Sep 16;17(9):e10105. doi: 10.15252/msb.202010105 (PMC8444284; doi:10.15252/msb.202010105)
Supplement: Supplementary file 5 — Source Data for Figure 3 [file MSB-17-e10105-s001.zip › Figure3A_sourcedata/GSEA_3065/hallmarks_state5.GseaPreranked.1623416302988/HALLMARK_EPITHELIAL_MESENCHYMAL_TRANSITION.html]

Details for gene set HALLMARK\_EPITHELIAL\_MESENCHYMAL\_TRANSITION[GSEA]

|  || Dataset | state5 |
| Phenotype | NoPhenotypeAvailable |
| Upregulated in class | na\_pos |
| GeneSet | HALLMARK\_EPITHELIAL\_MESENCHYMAL\_TRANSITION |
| Enrichment Score (ES) | 0.52195483 |
| Normalized Enrichment Score (NES) | 2.1566908 |
| Nominal p-value | 0.0 |
| FDR q-value | 0.0 |
| FWER p-Value | 0.0 |
Table: GSEA Results Summary

  

Fig 1: Enrichment plot: HALLMARK\_EPITHELIAL\_MESENCHYMAL\_TRANSITION      
 Profile of the Running ES Score & Positions of GeneSet Members on the Rank Ordered List

  

| PROBE | GENE SYMBOL | GENE\_TITLE | RANK IN GENE LIST | RANK METRIC SCORE | RUNNING ES | CORE ENRICHMENT || 1 | TNC |  |  | 45 | 0.680 | 0.0183 | Yes |
| 2 | COL4A1 |  |  | 54 | 0.634 | 0.0392 | Yes |
| 3 | CALU |  |  | 78 | 0.563 | 0.0559 | Yes |
| 4 | COL4A2 |  |  | 93 | 0.540 | 0.0729 | Yes |
| 5 | GPC1 |  |  | 98 | 0.530 | 0.0907 | Yes |
| 6 | TIMP1 |  |  | 109 | 0.518 | 0.1073 | Yes |
| 7 | ITGAV |  |  | 118 | 0.508 | 0.1239 | Yes |
| 8 | TFPI2 |  |  | 124 | 0.504 | 0.1407 | Yes |
| 9 | IGFBP2 |  |  | 137 | 0.491 | 0.1562 | Yes |
| 10 | PPIB |  |  | 156 | 0.477 | 0.1706 | Yes |
| 11 | ITGB1 |  |  | 157 | 0.477 | 0.1869 | Yes |
| 12 | SCG2 |  |  | 166 | 0.470 | 0.2022 | Yes |
| 13 | VCAN |  |  | 191 | 0.444 | 0.2148 | Yes |
| 14 | MATN2 |  |  | 213 | 0.431 | 0.2272 | Yes |
| 15 | SERPINE2 |  |  | 238 | 0.414 | 0.2387 | Yes |
| 16 | LRP1 |  |  | 259 | 0.404 | 0.2503 | Yes |
| 17 | CDH2 |  |  | 261 | 0.403 | 0.2641 | Yes |
| 18 | EMP3 |  |  | 264 | 0.400 | 0.2776 | Yes |
| 19 | PLOD3 |  |  | 270 | 0.398 | 0.2907 | Yes |
| 20 | CADM1 |  |  | 307 | 0.378 | 0.2997 | Yes |
| 21 | DST |  |  | 313 | 0.376 | 0.3120 | Yes |
| 22 | POSTN |  |  | 316 | 0.375 | 0.3247 | Yes |
| 23 | SPARC |  |  | 327 | 0.370 | 0.3363 | Yes |
| 24 | SGCB |  |  | 328 | 0.370 | 0.3490 | Yes |
| 25 | CD59 |  |  | 354 | 0.360 | 0.3585 | Yes |
| 26 | PTX3 |  |  | 355 | 0.360 | 0.3709 | Yes |
| 27 | CD44 |  |  | 378 | 0.349 | 0.3804 | Yes |
| 28 | LAMC1 |  |  | 382 | 0.348 | 0.3920 | Yes |
| 29 | COL5A2 |  |  | 407 | 0.337 | 0.4009 | Yes |
| 30 | SERPINH1 |  |  | 460 | 0.322 | 0.4061 | Yes |
| 31 | THY1 |  |  | 546 | 0.299 | 0.4068 | Yes |
| 32 | ADAM12 |  |  | 589 | 0.288 | 0.4120 | Yes |
| 33 | PLOD2 |  |  | 613 | 0.282 | 0.4191 | Yes |
| 34 | TNFRSF12A |  |  | 615 | 0.282 | 0.4287 | Yes |
| 35 | CDH11 |  |  | 630 | 0.279 | 0.4367 | Yes |
| 36 | PMP22 |  |  | 663 | 0.272 | 0.4424 | Yes |
| 37 | HTRA1 |  |  | 690 | 0.266 | 0.4486 | Yes |
| 38 | SPP1 |  |  | 724 | 0.256 | 0.4537 | Yes |
| 39 | FBLN1 |  |  | 743 | 0.252 | 0.4603 | Yes |
| 40 | NOTCH2 |  |  | 756 | 0.249 | 0.4676 | Yes |
| 41 | COLGALT1 |  |  | 768 | 0.248 | 0.4748 | Yes |
| 42 | PCOLCE |  |  | 773 | 0.247 | 0.4829 | Yes |
| 43 | NT5E |  |  | 834 | 0.235 | 0.4842 | Yes |
| 44 | P3H1 |  |  | 955 | 0.217 | 0.4781 | Yes |
| 45 | FSTL1 |  |  | 1086 | 0.200 | 0.4703 | Yes |
| 46 | QSOX1 |  |  | 1125 | 0.195 | 0.4727 | Yes |
| 47 | TGFBI |  |  | 1177 | 0.188 | 0.4734 | Yes |
| 48 | COPA |  |  | 1189 | 0.186 | 0.4786 | Yes |
| 49 | TIMP3 |  |  | 1191 | 0.186 | 0.4849 | Yes |
| 50 | SDC4 |  |  | 1193 | 0.186 | 0.4912 | Yes |
| 51 | ITGB5 |  |  | 1216 | 0.183 | 0.4950 | Yes |
| 52 | SERPINE1 |  |  | 1221 | 0.182 | 0.5008 | Yes |
| 53 | GPX7 |  |  | 1248 | 0.180 | 0.5040 | Yes |
| 54 | COL16A1 |  |  | 1291 | 0.173 | 0.5052 | Yes |
| 55 | COL5A1 |  |  | 1328 | 0.168 | 0.5069 | Yes |
| 56 | FN1 |  |  | 1342 | 0.167 | 0.5112 | Yes |
| 57 | LAMA1 |  |  | 1356 | 0.165 | 0.5154 | Yes |
| 58 | DKK1 |  |  | 1388 | 0.163 | 0.5175 | Yes |
| 59 | GJA1 |  |  | 1399 | 0.162 | 0.5220 | Yes |
| 60 | SDC1 |  |  | 1464 | 0.156 | 0.5201 | No |
| 61 | BMP1 |  |  | 1550 | 0.147 | 0.5156 | No |
| 62 | FUCA1 |  |  | 1611 | 0.141 | 0.5136 | No |
| 63 | VEGFA |  |  | 1634 | 0.139 | 0.5159 | No |
| 64 | COL5A3 |  |  | 1651 | 0.138 | 0.5188 | No |
| 65 | NTM |  |  | 1729 | 0.131 | 0.5147 | No |
| 66 | FBN1 |  |  | 1756 | 0.129 | 0.5162 | No |
| 67 | MMP2 |  |  | 1824 | 0.125 | 0.5129 | No |
| 68 | PLOD1 |  |  | 1919 | 0.117 | 0.5064 | No |
| 69 | LOXL2 |  |  | 2028 | 0.110 | 0.4979 | No |
| 70 | IGFBP3 |  |  | 2037 | 0.109 | 0.5008 | No |
| 71 | MEST |  |  | 2059 | 0.108 | 0.5021 | No |
| 72 | CXCL12 |  |  | 2165 | 0.101 | 0.4937 | No |
| 73 | GLIPR1 |  |  | 2261 | 0.094 | 0.4862 | No |
| 74 | SLC6A8 |  |  | 2268 | 0.094 | 0.4888 | No |
| 75 | EFEMP2 |  |  | 2557 | 0.076 | 0.4589 | No |
| 76 | COL6A2 |  |  | 2660 | 0.071 | 0.4498 | No |
| 77 | ECM1 |  |  | 2809 | 0.062 | 0.4353 | No |
| 78 | PLAUR |  |  | 2826 | 0.061 | 0.4356 | No |
| 79 | WNT5A |  |  | 2927 | 0.056 | 0.4262 | No |
| 80 | COL11A1 |  |  | 2950 | 0.055 | 0.4257 | No |
| 81 | ITGA5 |  |  | 3140 | 0.045 | 0.4059 | No |
| 82 | SNTB1 |  |  | 3353 | 0.034 | 0.3831 | No |
| 83 | APLP1 |  |  | 3430 | 0.030 | 0.3756 | No |
| 84 | TGFB1 |  |  | 3503 | 0.027 | 0.3684 | No |
| 85 | THBS1 |  |  | 3755 | 0.017 | 0.3407 | No |
| 86 | GAS1 |  |  | 3841 | 0.014 | 0.3316 | No |
| 87 | LOXL1 |  |  | 3988 | 0.007 | 0.3154 | No |
| 88 | CALD1 |  |  | 4714 | -0.022 | 0.2344 | No |
| 89 | BDNF |  |  | 4833 | -0.027 | 0.2220 | No |
| 90 | MCM7 |  |  | 4986 | -0.032 | 0.2060 | No |
| 91 | GADD45B |  |  | 5106 | -0.037 | 0.1938 | No |
| 92 | FGF2 |  |  | 5527 | -0.054 | 0.1483 | No |
| 93 | TPM2 |  |  | 5660 | -0.060 | 0.1355 | No |
| 94 | FERMT2 |  |  | 5664 | -0.061 | 0.1372 | No |
| 95 | ENO2 |  |  | 5895 | -0.069 | 0.1137 | No |
| 96 | CAP2 |  |  | 6013 | -0.075 | 0.1030 | No |
| 97 | SFRP1 |  |  | 6042 | -0.076 | 0.1025 | No |
| 98 | NNMT |  |  | 6137 | -0.082 | 0.0947 | No |
| 99 | RHOB |  |  | 6287 | -0.090 | 0.0810 | No |
| 100 | PTHLH |  |  | 6317 | -0.091 | 0.0808 | No |
| 101 | WIPF1 |  |  | 6390 | -0.095 | 0.0760 | No |
| 102 | TAGLN |  |  | 6733 | -0.116 | 0.0414 | No |
| 103 | FLNA |  |  | 6856 | -0.126 | 0.0319 | No |
| 104 | MSX1 |  |  | 6868 | -0.127 | 0.0351 | No |
| 105 | CAPG |  |  | 6974 | -0.135 | 0.0279 | No |
| 106 | PRRX1 |  |  | 7057 | -0.142 | 0.0235 | No |
| 107 | MYLK |  |  | 7203 | -0.155 | 0.0125 | No |
| 108 | ID2 |  |  | 7333 | -0.171 | 0.0038 | No |
| 109 | GADD45A |  |  | 7633 | -0.208 | -0.0227 | No |
| 110 | PMEPA1 |  |  | 7830 | -0.241 | -0.0366 | No |
| 111 | JUN |  |  | 7930 | -0.262 | -0.0387 | No |
| 112 | TPM1 |  |  | 8012 | -0.278 | -0.0383 | No |
| 113 | SAT1 |  |  | 8162 | -0.314 | -0.0444 | No |
| 114 | PFN2 |  |  | 8545 | -0.455 | -0.0718 | No |
| 115 | PDLIM4 |  |  | 8630 | -0.512 | -0.0637 | No |
| 116 | VIM |  |  | 8663 | -0.535 | -0.0489 | No |
| 117 | DPYSL3 |  |  | 8692 | -0.554 | -0.0330 | No |
| 118 | TPM4 |  |  | 8905 | -0.778 | -0.0302 | No |
| 119 | LGALS1 |  |  | 8982 | -1.135 | 0.0002 | No |
Table: GSEA details [plain text format]

  

Fig 2: HALLMARK\_EPITHELIAL\_MESENCHYMAL\_TRANSITION: Random ES distribution      
 Gene set null distribution of ES for **HALLMARK\_EPITHELIAL\_MESENCHYMAL\_TRANSITION**
